# Acupuncture for postoperative pain of lumbar disc herniation: A systematic review and meta-analysis

**DOI:** 10.1097/MD.0000000000032016

**Published:** 2022-12-09

**Authors:** Weidong Zhang, Huan Liu, Xuezhen Le, Kunyu Song, Fo Yang, Zhenhai Cui, Wenhai Zhao

**Affiliations:** a Changchun University of Chinese Medicine, Changchun, China; b Nanchang Hongdu Hospital of Traditional Chinese Medicine, Nanchang, China; c The Third Affiliated Hospital of Changchun University of Chinese Medicine, Changchun, China; d Affiliated Hospital of Changchun University of Chinese Medicine, Changchun, China.

**Keywords:** acupuncture, lumbar disc herniation, postoperative pain, systematic review

## Abstract

Evidence for the effects of acupuncture on postoperative pain of Lumbar disc herniation (LDH) is inconsistent. We conducted this systematic review and meta-analysis to evaluate the efficacy of acupuncture for postoperative pain of LDH.

**Methods:**

We performed a comprehensive search on PubMed, Embase, Web of Science, Cochrane Central, and four Chinese databases (CNKI, Wan Fang, CBMdisc, and VIP) for articles published before September 2021. The data extraction table was made for the ten included studies, and the risk of bias was assessed using the Cochrane collaboration tool, followed by statistical analysis using RevMan 5.3.

**Results:**

A total of ten studies involving 838patients were included. The statistical meta-analysis showed that acupuncture treatment was significantly better than drugs in improving cure rate (OR = 2.24, 95% CI = [1.58, 3.19], *P* < .00001) and the total effectiveness rate (OR = 4.85, 95% CI = [2.59, 9.08], *P* < .00001). And the results from the meta-analysis showed that acupuncture group was superior to control group in debasing visual analog scale score (MD = −1.26, 95% CI = [−1.72, −0.79], *P* < .00001) and improving Japanese Orthopaedic Association score (MD = 4.21, 95% CI = [1.53, 6.90], *P* < .00001). In addition, acupuncture was statistically significantly better than drugs (OR = 0.27, 95% CI = [0.11, 0.62], *P* = .002) in the incidence of adverse events, However, there was no statistically significant difference between acupuncture and rehabilitation (OR = 0.36, 95% CI = [0.07, 1.98], *P* = .24).

**Conclusion:**

Acupuncture is an effective and safe treatment for postoperative pain of LDH. It can be recommended to manage patients with postoperative pain of LDH. However, considering the unsatisfactory quality of the included studies, more high-quality randomized controlled trials with a large sample size are needed to elucidate this issue.

## 1. Introduction

Lumbar disc herniation (LDH) is a common degenerative spinal disease, which is one of the leading causes of low back pain (LBP).^[1]^ Nearly 80% of the Western population sustains an episode of LBP at least once during their lifetime.^[2,3]^ The incidence of LBP is higher in females, especially between the ages 40 to 69 years.^[4]^ Sciatica (low back-related leg pain) is one of the most typical symptoms of LBP. Nonetheless, LBP and sciatica represent symptoms rather than specific diagnoses. In a wide range of differentiation, the most common source is intervertebral degeneration leading to degenerative disc disease and LDH.^[5]^ The main signs and symptoms of LDH are radicular pain, paresthesia, and weakness in the distribution of one or more lumbosacral nerve roots. The actual event leading to disc herniation remains unclear.^[6,7]^ In the case of the herniated intervertebral disc, nucleus pulposus tissue protrudes through the ruptured annulus fibrosus, which subsequently impacts the spinal nerve roots and causes LBP or radiating leg pain.^[8]^ The initial clinical treatment of LDH is generally conservative. Most of them are drugs, acupuncture, physical therapy, massage, etc., which have significant effects, but the treatment cycle is longer. Patients with lumbar intervertebral disc herniation are more severe in the later stage and are mostly treated with surgery, which can significantly improve patients’ clinical symptoms. However, some patients still have a poor prognosis, residual pain, and short-term postoperative recurrence of symptoms, which seriously affect the patient’s postoperative recovery.^[9,10]^ Therefore, effectively improving patients’ postoperative pain with LDH has become a significant clinical problem.

Acupuncture, a standard nonpharmacological treatment by stimulating specific acupoints on the body, has been widely used for various diseases, especially those involving multiple painful disorders.^[11,12]^ Acupuncture can regulate neuroendocrine function by stimulating acupoints, such as increasing the production of endogenous opioid peptides to achieve analgesic effects.^[13]^ Allodynia is a common symptom in patients with neuropathic pain. Acupuncture activates the local molecular signaling pathway components, mainly extracellular signal-regulated kinase, thereby attenuating nociceptive behavior and reducing mechanical allodynia.^[14]^ In many published clinical studies on acupuncture for postoperative pain of LDH, most studies have shown that acupuncture is a reliable treatment for postoperative pain of LDH.

There has been a systematic review of acupuncture for LDH.^[15]^ However, there is still a lack of periodic review of acupuncture for postoperative pain of LDH, and the effectiveness is unclear. Therefore, we performed a systematic review and meta-analysis to assess the strength of the current evidence to support the efficacy and safety of acupuncture for postoperative pain of LDH.

## 2. Methods

This article was performed according to the Preferred Reporting Items for Systematic Review and Meta-Analysis (PRISMA) statement.^[16]^

### 2.1. Search strategy

Two reviewers (W.Z. and H.L.) independently conducted a comprehensive literature search from multiple electronic databases starting September 1, 2021, including PubMed, Embase, Web of Science, Cochrane Central, and four Chinese databases (CNKI, Wan Fang, CBMdisc, and VIP). We included only randomized controlled trials (RCTs) that used acupuncture therapy as the primary treatment for postoperative pain of LDH. The search keywords used were Postoperative Pain of Lumbar Disc Herniation,” Lumbar Disc Herniation,” lumbar discopathy,” Acupuncture,” Acupoints,” Needle,” as well as random,” clinical,” and there was no restriction on language. Then, the two reviewers browsed the abstract and the complete text, respectively, and selected eligible studies that met the inclusion criteria. In addition, all available studies related to postoperative pain of LDH treatment were manually checked for any additional RCTs that may be relevant.

### 2.2. Inclusion and exclusion criteria

Relevant studies were included if they met the following criteria: the included trials were RCTs investigating acupuncture for postoperative pain of LDH; the included patients were diagnosed with postoperative LDH, regardless of the surgical method, their nationality, race, gender, or age; the primary outcome measures included Chinese medical efficacy criteria (including cure rate and the total effectiveness rates), Japanese Orthopaedic Association Scores(JOA), and visual analog scale (VAS); the secondary outcome was adverse events to assess safety; and the full text should be provided. Studies were excluded if any of the following existed: animal studies, case reports, reviews, and non-RCTs.

### 2.3. Data extraction

Two reviewers (M.J. and X.L.) independently extracted relevant data from the included studies. Essential information mainly had first author, publication year, study design, sample size, intervention duration, control group, outcome measures, and adverse events. Finally, cross-check. We will solve any uncertainty through discussion or consulting other reviewers (F.Y. and K.S.) if there is any uncertainty. If there is any unclear information in some studies, we try to contact the first author for detailed information.

### 2.4. Quality assessment

Two reviewers (Z.L. and S.L.) used the Cochrane risk of bias (ROB) tool to independently assess the included studies’ quality and ROB. The content consists of random sequence generation, allocation concealment, blinding of participants and personnel, blinding of outcome evaluation, incomplete outcome data, selective report, and other sources of bias. Disagreements will be resolved through discussion.

### 2.5. Statistical analysis

Meta-analysis was performed using Reviewer Manager software version 5.3. For continuous variables (JOA and VAS), we estimated the combined mean difference (MD) with 95% confidence intervals (CI); for categorical data (total effectiveness rates, cure rate, and adverse events), we calculated pooled odds ratios (OR) with 95% CI. The Higgins *I*^2^ test evaluated the heterogeneity of the studies. The fixed-effect model was used when *I*^2^ ≤ 50%; otherwise, the random effect model was applied. Sensitivity analyses were used to assess the impact of all included studies on the final results. If *P* < .05, this was considered statistically significant between studies.

## 3. Results

### 3.1. Literature search results

**Figure 1.**
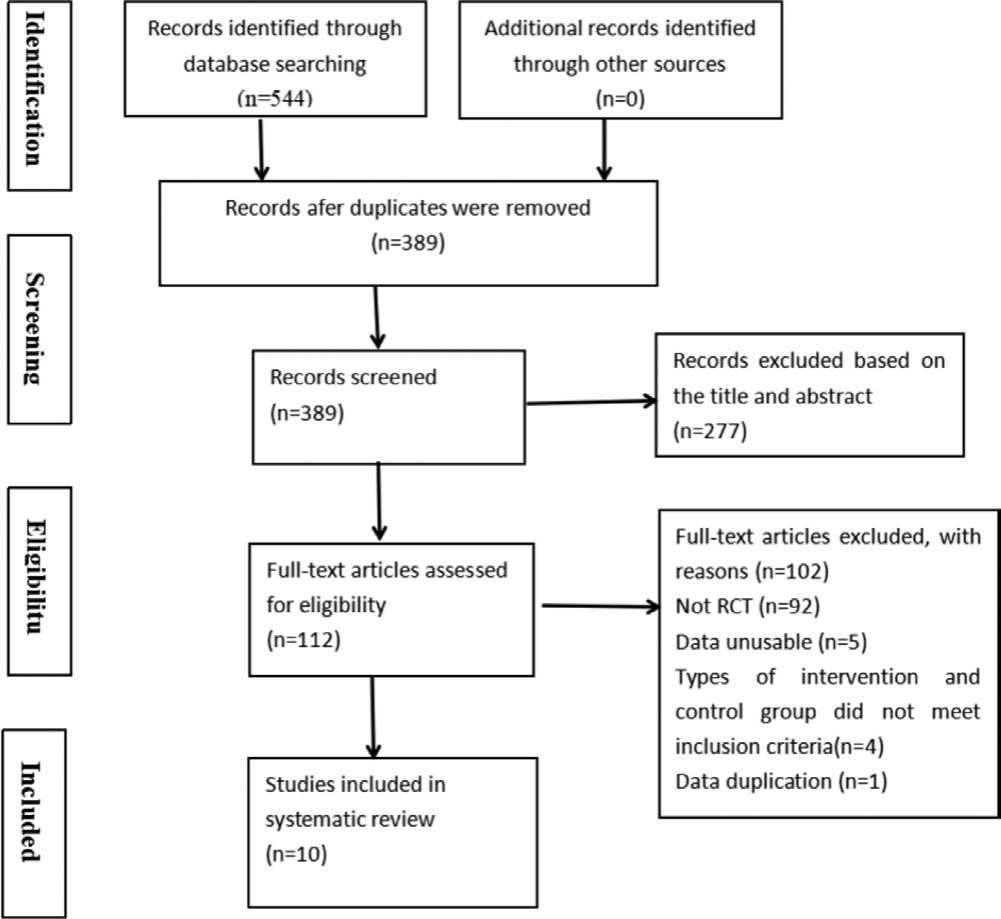
Flow diagram for study selection. RCTs = randomized controlled trials.

Our search of the above database using keywords yielded 544 studies based on the retrieval strategy. Three hundred eighty-nine studies remained after excluding 155 duplicates with EndNote X9 software. After reviewing the titles and abstracts,379 were eliminated for various reasons, including incomplete data, irrelevant content, and non-RCTs. Finally, 10 RCTs^[17–26]^ met our inclusion criteria after reviewing the entire text carefully. A flow diagram depicting the whole study selection process is shown in Figure 1.

### 3.2. Study characteristics

Table 1 describes the essential elements of the included studies. All included studies were single-center RCTs published between 2006 and 2021. The sample sizes ranged from 53 to 110. In total, 838 patients (423 in the acupuncture group, 415 in the control group) treated with acupuncture for postoperative pain of LDH were included in the meta-analysis. In 10 studies, all research teams were from China.^[17–26]^ Nine studies^[18–26]^ compared acupuncture with drugs, and one^[17]^ compared acupuncture with rehabilitation training.

**Table 1:** Characteristics of the included RCTs.

Study ID	Sample size (T/C)	Intervention		Frequency of acupuncture	Duration of acupuncture, d	Outcomes
		Treatment group	Control group			
Jiang 2019	31/30	Acupuncture	Drugs	Once a day	14	①②③④ ⑤
Li 2021	28/25	Acupuncture	Drugs	Once a day	14	③④
Li 2006	40/37	Acupuncture	Drugs	Once a day	14	①②③
Wang 2018	42/42	Acupuncture	Drugs	Once 2 days	28	①②
Chen 2019	50/50	Acupuncture	Drugs	Once a day	14	①②③④
Wang 2015	34/34	Acupuncture	Drugs	Once a day	15	①②④
Xia 2018	55/55	Acupuncture	Drugs	Once a day	15	①②④
Deng 2017	42/42	Acupuncture	Drugs	Twice a day	14	①②④
Yu 2019	54/54	Acupuncture	Drugs	Once a day	15	①②③④⑤
Ye 2021	47/46	Acupuncture	Rehabilitation training	Once a day	28	③④⑤

① Effective rate, ② Cure rate, ③ JOA, ④ VAS, ⑤ Adverse.

JOA = Japanese Orthopaedic Association, RCTs = randomized controlled trials, VAS = visual analog scale.

### 3.3. Quality assessment

**Figures 2.**
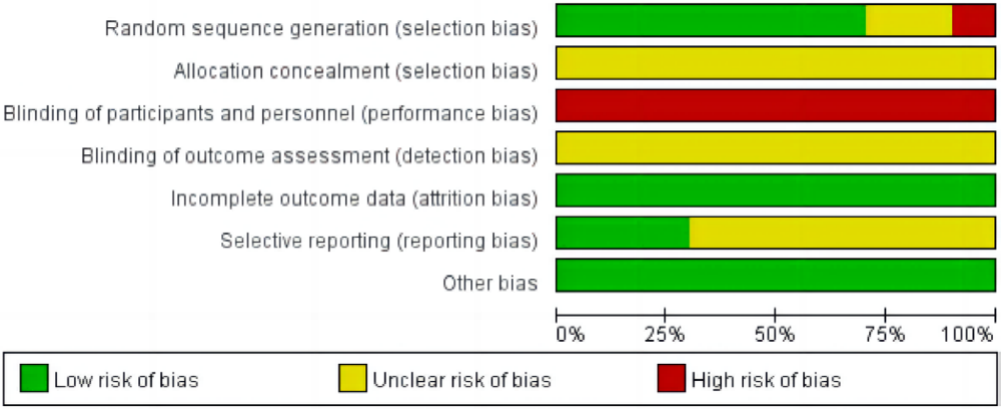
Risk of bias graph.

**Figures 3.**
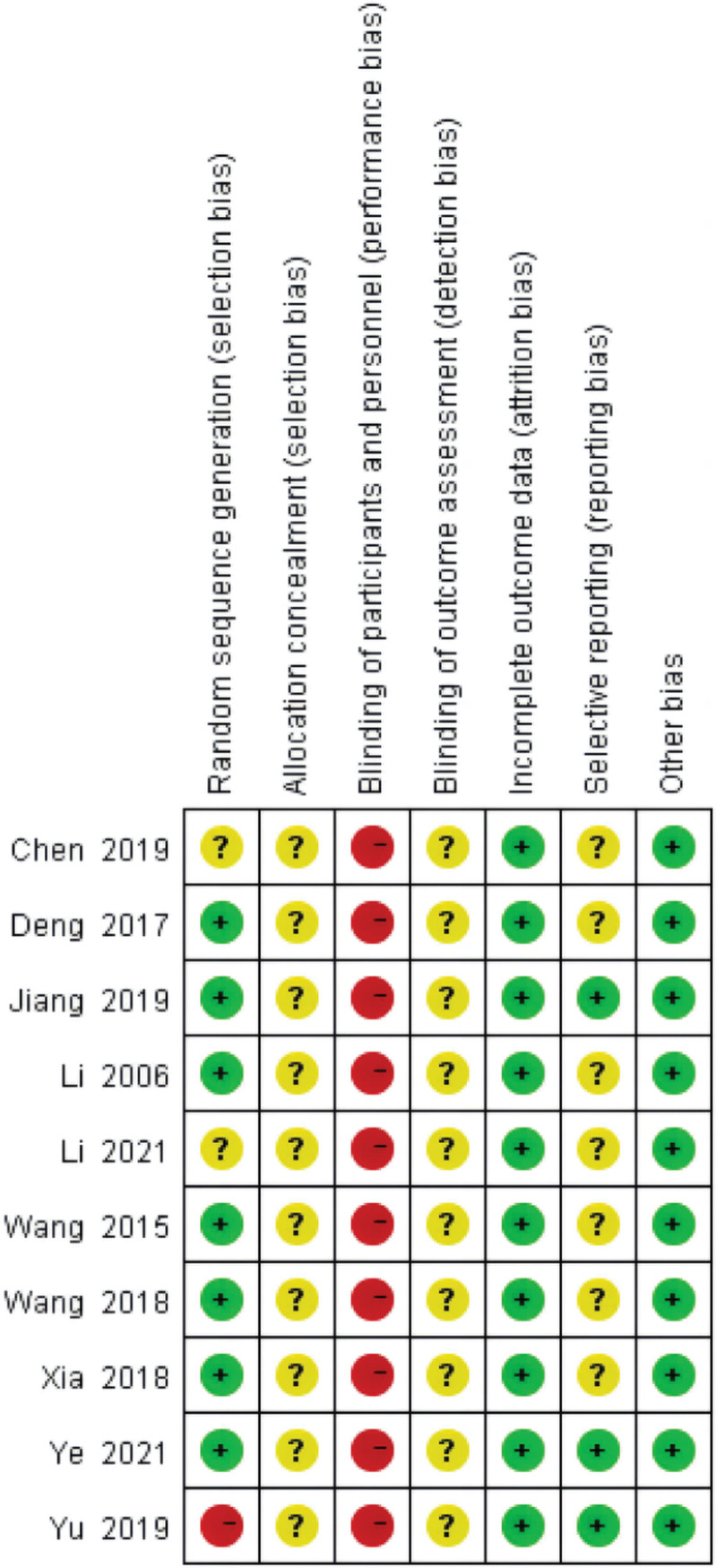
Risk of bias summary.

A summary of the ROB in the included trials is shown in Figures 2 and 3. Six studies^[17,18,21,23–25]^ used a random number table for generation random sequence, one^[20]^ used coin tossing, two studies^[19,22]^ only mentioned random,” and one^[26]^ grouped by treatment regimen to generate a random sequence. No study found any details in the domains of accidental allocation concealment and selective outcome reporting. It is tough to blind acupuncture therapists with blinding of participants and personnel. Two studies^[17,18]^ reported the number and reasons for dropouts, and other studies reported no missing data. Three studies^[17,18,26]^ reported detailed information about adverse events.

### 3.4. Cure rate

**Figure 4.**
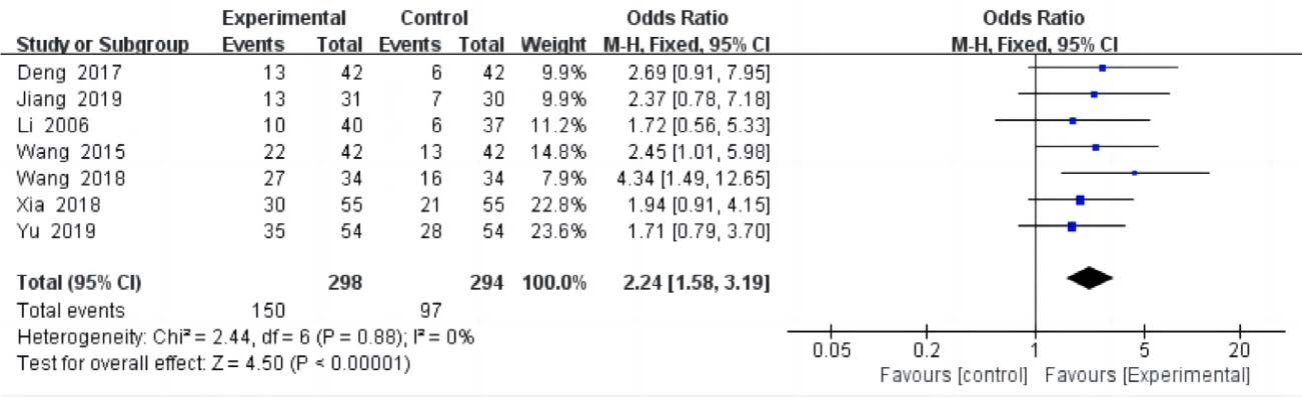
The cure rate. CI = confidence interval.

Seven studies^[18,20,21,23–26]^ analyzed the cure rate of acupuncture for postoperative pain of LDH, and all compared acupuncture with drugs. No heterogeneity (*P* = .88, *I*^2^ = 0%) was found in cure rates. The results from our meta-analysis with a fixed-effects model showed that acupuncture was higher than drugs (OR = 2.24, 95% CI = [1.58, 3.19], *P* < .00001) in improving cure rate (Fig. 4).

### 3.5. The total effectiveness rate

**Figure 5.**
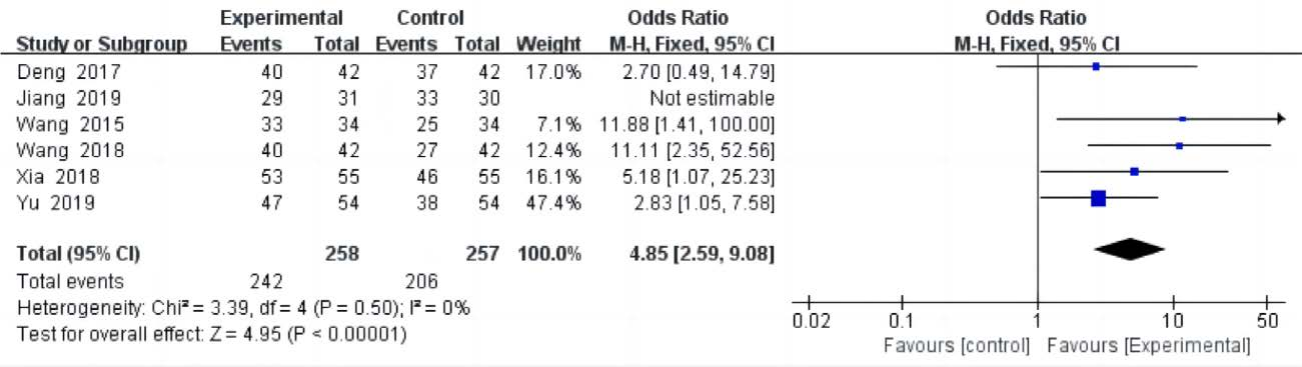
The effective rate. CI = confidence interval.

Six studies^[18,19,21,23,25,26]^ analyzed the total effective rate of acupuncture for postoperative pain of LDH, and all compared acupuncture with drugs. No heterogeneity (*P* = .50, *I*^2^ = 0%) was found in the total effectiveness rates. The results from our meta-analysis with a fixed-effects model showed that acupuncture was higher than drugs (OR = 4.85, 95% CI = [2.59, 9.08], *P* < .00001) in improving the total effectiveness rate (Fig. 5).

### 3.6. VAS score

**Figure 6.**
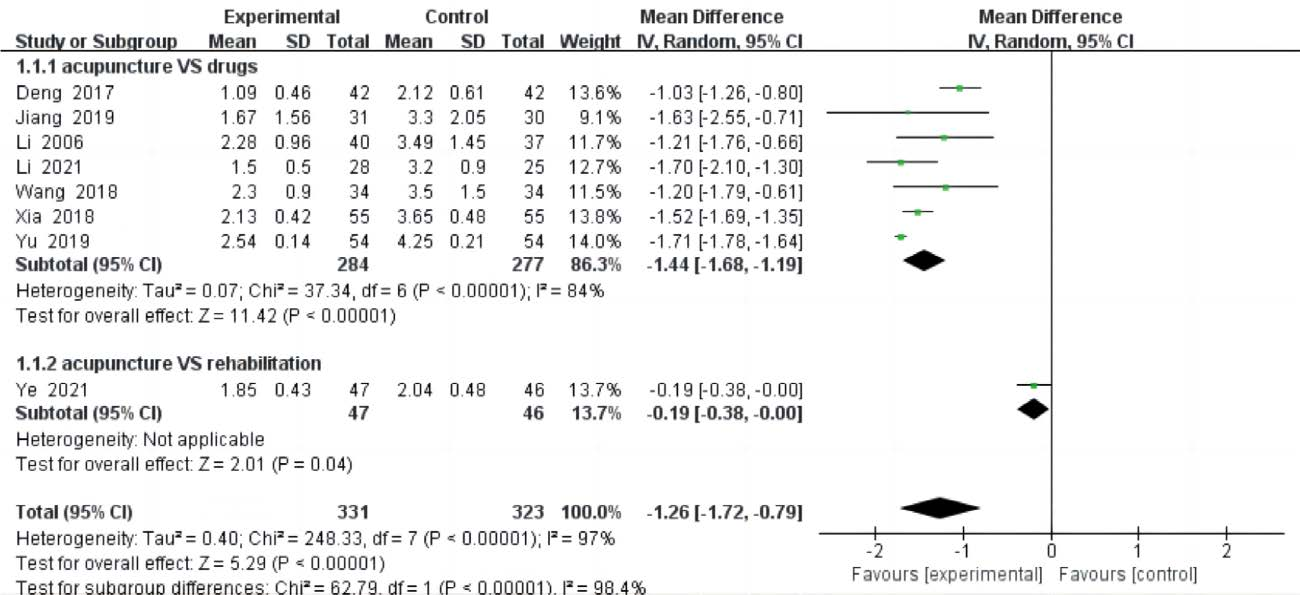
VAS score. CI = confidence interval, SD = standard deviation, VAS = visual analog scale.

Eight reviews^[17–21,24–26]^ comparing the effects of acupuncture with those of drugs and rehabilitation used the VAS score. Seven studies^[18–21,24–26]^ compared acupuncture with drugs, and one^[17]^ compared acupuncture with rehabilitation. The data results revealed a high degree of heterogeneity among these RCTs (*P* < .00001, *I*^2^ = 98.4%). The results from our meta-analysis with a random-effects model showed that acupuncture was better than the control group (MD = −1.26, 95% CI = [−1.72, −0.79], *P* < .00001) in decreasing VAS score. The subgroup analysis exploring the improvement in the VAS score among different control groups showed that acupuncture is statistically significantly better than drugs (MD = −1.44, 95% CI = [−1.68, −1.99], *P* < .00001, heterogeneity: *P* < .00001, *I*^2^ = 84%) and rehabilitation (MD = −0.19, 95% CI = [−0.38, −0.00], *P* < .00001) (Fig. 6).

### 3.7. JOA score

**Figure 7.**
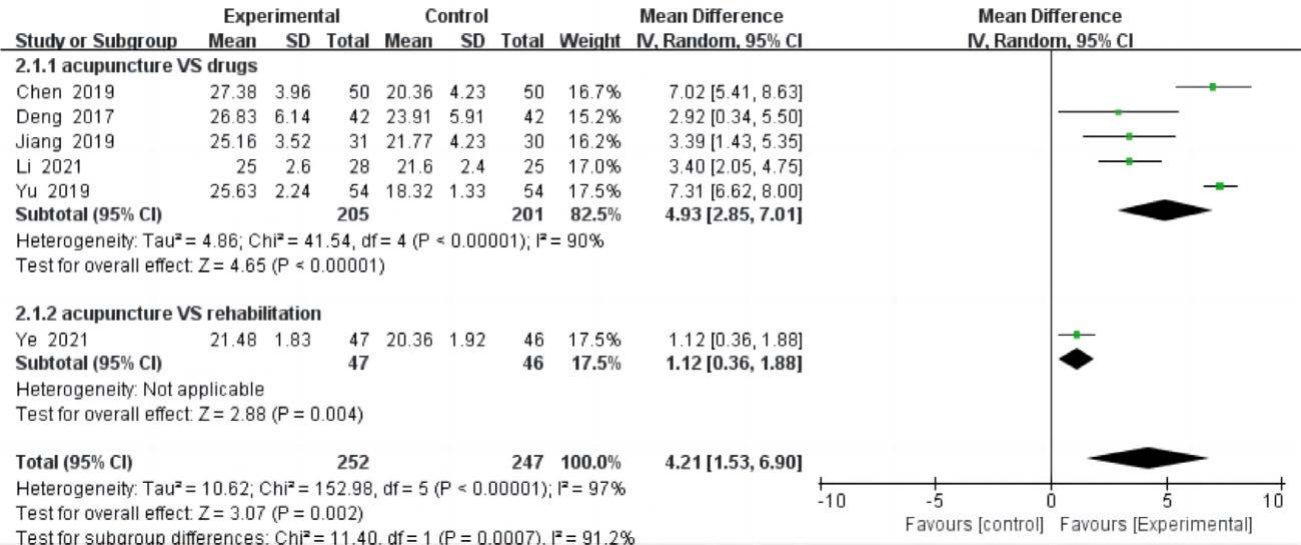
JOA score. CI = confidence interval, JOA = Japanese Orthopaedic Association, SD = standard deviation.

Six reviews^[17–19,22,25,26]^ comparing the effects of acupuncture with those of drugs and rehabilitation used the JOA score. Five studies^[18,19,22,25,26]^ compared acupuncture with drugs, and one study^[17]^ compared acupuncture with rehabilitation. The data results revealed a high degree of heterogeneity among these RCTs (*P* = .0007, *I*^2^ = 91.2%). The results from our meta-analysis with a random-effects model showed that acupuncture was higher than the control group (MD = 4.21, 95% CI = [1.53, 6.90], *P* < .00001) in improving the JOA score. The subgroup analysis exploring the improvement in the VAS score among different control groups showed that acupuncture is statistically significantly better than drugs (MD = 4.93, 95% CI = [2.85, 7.01], *P* < .00001, heterogeneity: *P* < .00001, *I*^2^ = 90%) and rehabilitation (MD = 1.12, 95% CI = [0.36, 1.88], *P* = .004) (Fig. 7).

### 3.8. Adverse events

**Figure 8.**
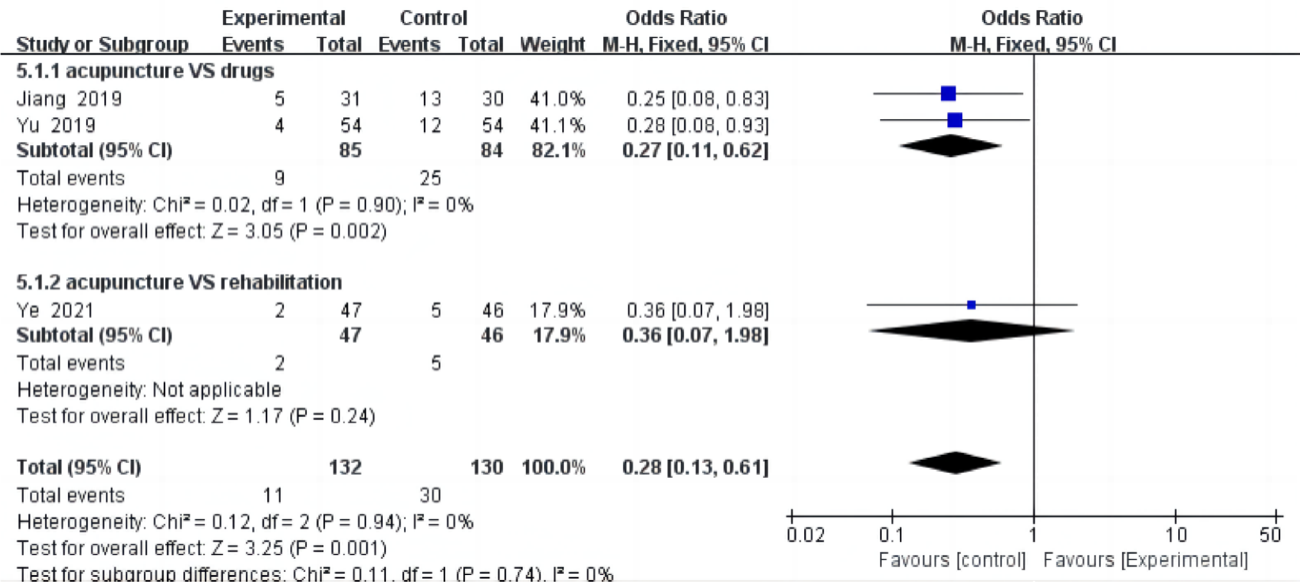
Adverse events. CI = confidence interval.

Three included studies^[17,18,26]^ reported adverse events, describing the number of specific adverse events occurring. The adverse events mentioned in those studies were mainly aggravated pain, urinary retention, postoperative infection, lower limb venous thrombosis, etc. No heterogeneity (*P* = .94, *I*^2^ = 0%) was found in adverse events. The results from our meta-analysis with a fixed-effects model showed that it was not statistically different from the control group (OR = 0.28, 95% CI = [0.13, 0.61], *P* = .74) in adverse events. However, the subgroup analysis showed that acupuncture was statistically significantly better than drugs (OR = 0.27, 95% CI = [0.11, 0.62], *P* = .002) in the incidence of adverse events (Fig. 8).

### 3.9. Publication bias

Publication bias could not be analyzed adequately because no meta-analysis involving more than ten studies was performed.

## 4. Discussion

LDH is a condition that tremendously affects one’s quality of life. With the notable increase in the aged population and the high work pressure and lifestyle changes in young people, the incidence of LDH is gradually increased.^[27]^ With the continuous improvement of medical technology and the high requirements of people for rapidly improving pain and improving quality of life, surgery treatment for LDH has gradually become the choice of increasing patients, such as discectomy laminoplasty and nucleosome, etc.^[28,29]^ Surgical therapy is an effective therapy that can effectively relieve the compression of the spinal cord and nerves by the intervertebral disc and rapidly relieve clinical symptoms such as pain and numbness. However, long-term follow-up revealed that most patients had complications such as postoperative pain recurrence, limited motor function, poor spinal stability, and scar adhesion caused by local tissue of the surgical rupture ring, which aggravated the patient’s pain symptoms to a certain extent, and the late satisfaction evaluation was low.^[10,30,31]^

Acupuncture, as a part of traditional Chinese medicine, has a good analgesic effect, and it is easy to operate, economical, and has few side effects.^[32]^ Acupuncture has been widely recognized globally, such as in the United States, the United Kingdom, and Canada. In the United States, acupuncture is one of the traditional medical treatments with a national licensure system.^[33]^ The local effects of acupuncture include reducing inflammatory mediators such as substance P, TNF-α, IL-1β, IL-8, and IL-10. Segmental inhibition, the release of endogenous opioid, 5-HT, adrenergic, and NMDA/AMPA/KA pathways to produce analgesic effects and reduce central sensitization.^[14,34]^ At the same time, studies have shown that acupuncture can cause transient blood flow changes in the nerve structure, regulate the function of the endogenous pain regulation network, and lower plasma adrenocorticotrophic hormone levels. In addition, acupuncture can improve the blood circulation and oxygen supply of the cauda equina, nerve root, and sciatic nerve, promote nerve recovery, and improve symptoms.^[35–37]^ These factors may help explain the sound effect of acupuncture for postoperative pain of LDH.

Our study collected and analyzed data from ten studies involving 838 patients. Our Comprehensive analysis indicated that acupuncture treatment was significantly better than drug and rehabilitation in improving the total effective rate and cure rate. In terms of JOA and VAS scores, acupuncture showed a more favorable effect on relieving pain than drugs and rehabilitation. In this meta-analysis, only three studies reported related adverse events. Our analysis of relevant data shows that acupuncture is superior to medications in the incidence of adverse events, while acupuncture and rehabilitation are not statistically significant. Therefore, acupuncture has fewer adverse effects than drugs, and we recommend acupuncture for LDH postoperative pain. At the same time, in the process of acupuncture operation, it is necessary to strengthen the aseptic operation specification and improve the professional ability of doctors, which can effectively avoid the occurrence of these adverse events. Above, according to the findings of our analysis, we believe that acupuncture is an effective and safe treatment for postoperative pain of LDH.

## 5. Limitations

Our study has limitations. First, the overall methodological quality of the RCTs included was moderate, with only 6 studies describing the random method, no studies mentioning the use of allocation concealment, and no placebo-controlled studies. Second, small sample size and the insufficient number of included studies may lead to inaccurate evidence in our research. Third, all included studies were conducted in China, and the results may vary according to geographic region or ethnicity. Last, the total effective rate and cure rate was used as the outcome measurement in most of the included RCTs, but this is not an internationally recognized outcome.

## 6. Conclusion

Our systematic review and meta-analysis revealed that acupuncture could reduce the postoperative pain of LDH. In terms of safety, the incidence of adverse events of acupuncture is significantly lower than that of drugs. However, more high-quality RCTs with a lower ROB and adequate sample sizes are needed to investigate further the therapeutic effect of acupuncture for postoperative pain of LDH.

## Author contributions

**Data curation:** Meiying Jin, Xuezhen Le.

**Formal analysis:** Kunyu Song, Zongyang Li.

**Project administration:** Shubo Li, Fo Yang.

**Software:** Longhao Wei, Zhenhai Cui.

**Writing – original draft:** Weidong Zhang, Huan Liu, Liquan Sha.

**Writing – review & editing:** Weidong Zhang, Huan Liu, Wenhai Zhao.

## References

[1] Hoy D March L Brooks P. The global burden of low back pain: estimates from the Global Burden of Disease 2010 study. Ann Rheum Dis. 2014;73:968–74. 24665116 10.1136/annrheumdis-2013-204428

[2] Andersson GB. Epidemiological features of chronic low-back pain. Lancet. 1999;354:581–5. 10470716 10.1016/S0140-6736(99)01312-4

[3] Patrick N Emanski E Knaub MA. Acute and chronic low back pain. Med Clin North Am. 2016;100:169–81. 26614726 10.1016/j.mcna.2015.08.015

[4] Maher C Underwood M Buchbinder R. Non-specific low back pain. Lancet. 2017;389:736–47. 27745712 10.1016/S0140-6736(16)30970-9

[5] Wang LL Fan WQ Yu CH. Clinical effects of electrical stimulation therapy on lumbar disc herniation-induced sciatica and its influence on peripheral ROS level. J Musculoskelet Neuronal Interact. 2018;18:393–8. 30179218 PMC6146184

[6] Vroomen PC Krom M.C. de Wilmink JT. Diagnostic value of history and physical examination in patients suspected of lumbosacral nerve root compression. J Neurol Neurosurg Psychiatry. 2002;72:630–4. 11971050 10.1136/jnnp.72.5.630 PMC1737860

[7] Vucetic N Svensson O. Physical signs in lumbar disc hernia. Clin Orthop Relat Res. 1996;333:192–201. 8981896

[8] DiStefano TJ Shmukler JO Danias G. The functional role of interface tissue engineering in annulus fibrosus repair: bridging mechanisms of hydrogel integration with regenerative outcomes. ACS Biomater Sci Eng. 2020;6:6556–86. 33320618 10.1021/acsbiomaterials.0c01320 PMC7809646

[9] Ulutaş M ÇInar K Seçer M. The surgery and early postoperative radicular pain in cases with multifocal lumbar disc herniation. Medicine (Baltim). 2017;96:e6238. 10.1097/MD.0000000000006238 PMC5340462 28248889

[10] Bilgin E Ökten A. Post-operative pain management for single-level lumbar disc herniation surgery: a comparison of betamethasone, ibuprofen, and pregabalin. Agri. 2021;33:89–95. 33913134 10.14744/agri.2020.82335

[11] Bauer BA Tilburt JC Sood A. Complementary and alternative medicine therapies for chronic pain. Chin J Integr Med. 2016;22:403–11. 27339090 10.1007/s11655-016-2258-y

[12] Birch S Bovey M Alraek T. Acupuncture as a treatment within integrative health for palliative care: a brief narrative review of evidence and recommendations. J Altern Complement Med. 2020;26:786784–793. 10.1089/acm.2020.0032 32924554

[13] Harris RE Zubieta JK Scott DJ. Traditional Chinese acupuncture and placebo (sham) acupuncture are differentiated by their effects on mu-opioid receptors (MORs). Neuroimage. 2009;47:1077–85. 19501658 10.1016/j.neuroimage.2009.05.083 PMC2757074

[14] Lai HC Lin YW Hsieh CL. Acupuncture-analgesia-mediated alleviation of central sensitization. Evid Based Complement Alternat Med. 2019;2019:6173412. 30984277 10.1155/2019/6173412 PMC6431485

[15] Tang S Mo Z Zhang R. Acupuncture for lumbar disc herniation: a systematic review and meta-analysis. Acupunct Med. 2018;36:62–70. 29496679 10.1136/acupmed-2016-011332

[16] Moher D Liberati A Tetzlaff J PRISMA Group. Preferred reporting items for systematic reviews and meta-analyses: the PRISMA statement. BMJ. 2009;339:b2535. 19622551 10.1136/bmj.b2535 PMC2714657

[17] Ye BH Ye L Mao XY. Observation on effects of needle warming moxibustion combined with rehabilitation training on recovery process of patients with lumbar disc herniation after percutaneous endoscopic lumbar discectomy. Chin Arch Trad Chin Med. 2022;40:212–5.

[18] Jiang Y. Observation on the therapeutic effect of warm needle moxibustion on postoperative pain of lumbar disc herniation. Pract Clin J Int Trad Chin West Med. 2019;19:79–80.

[19] Li Z. Clinical observation of warm needle moxibustion for postoperative pain of lumbar disc herniation. Chin Naturop. 2021;29:49–52.

[20] Li XQL. Acupuncture mainly treats the residual pain in the recovery period of lumbar disc herniation. Chin Acupunc Moxib. 2006;1:566–8.

[21] Wang M. Therapeutic effect of acupuncture on residual lumbago and leg pain after lumbar disc herniation surgery. Clin Med. 2018;38:118–21.

[22] Chen TL Lian ZR Chen BL. Clinical study on acupuncture treatment of residual symptoms after lumbar disc herniation surgery. Shaanxi J Trad Chin Med. 2019;40:1138–40.

[23] Wang F. Acupuncture treatment of 34 cases of postoperative pain of lumbar intervertebral disc herniation. Clin J Chin Med. 2015;7:21–2.

[24] Xia SY. Acupuncture treatment of 55 cases of postoperative pain of lumbar disc herniation. Guangming J Chin Med. 2018;33:1446–8.

[25] Deng YD. Observation on therapeutic effect of acupuncture on postoperative pain of lumbar disc herniation. World Chin Med. 2017;12:639–42.

[26] Yu J. Analysis of clinical effect of acupuncture on postoperative pain of lumbar disc herniation. China J Pharma Econ. 2019;14:55–7.

[27] Aizawa T Kokubun S Ozawa H. Increasing incidence of degenerative spinal diseases in Japan during 25 years: the registration system of spinal surgery in Tohoku University Spine Society. Tohoku J Exp Med. 2016;238:153–63. 26876801 10.1620/tjem.238.153

[28] Sairyo K Chikawa T Nagamachi A. State-of-the-art transforaminal percutaneous endoscopic lumbar surgery under local anesthesia: discectomy, foraminoplasty, and ventral facetectomy. J Orthop Sci. 2018;23:229–36. 29248305 10.1016/j.jos.2017.10.015

[29] Kamper SJ Ostelo R Rubinstein SM. Minimally invasive surgery for lumbar disc herniation: a systematic review and meta-analysis. Eur Spine J. 2014;23:1021–43. 24442183 10.1007/s00586-013-3161-2

[30] Yan DL Zhang ZH Zhang Z. Residual leg numbness after endoscopic discectomy treatment of lumbar disc herniation. BMC Musculoskelet Disord. 2020;21:273. 32340609 10.1186/s12891-020-03302-5 PMC7187494

[31] Shenoy K Stekas N Donnally CJ. Retrolisthesis and lumbar disc herniation: a postoperative assessment of outcomes at 8-year follow-up. Spine J. 2019;19:995–1000. 30594668 10.1016/j.spinee.2018.12.010

[32] Li Y Wu F Cheng K. [Mechanisms of acupuncture for inflammatory pain]. Zhen Ci Yan Jiu. 2018;43:467–75. 30232847 10.13702/j.1000-0607.180196

[33] Lim TK Ma Y Berger F. Acupuncture and neural mechanism in the management of low back pain-an update. Medicines (Basel). 2018;5:63. 29941854 10.3390/medicines5030063 PMC6164863

[34] Zhang R Lao L Ren K. Mechanisms of acupuncture-electroacupuncture on persistent pain. Anesthesiology. 2014;120:482–503. 24322588 10.1097/ALN.0000000000000101 PMC3947586

[35] Inoue M Kitakoji H Yano T. Acupuncture treatment for low back pain and lower limb symptoms-the relation between acupuncture or electroacupuncture stimulation and sciatic nerve blood flow. Evid Based Complement Alternat Med. 2008;5:133–43. 18604251 10.1093/ecam/nem050 PMC2396470

[36] Ma WZ Zhang YM Yuan H. [Effects of balance-acupuncture stimulation of ‘back pain’ and ‘hip pain’ points on plasma beta-endorphin and ACTH contents in rats with lumbar disc herniation]. Zhen Ci Yan Jiu. 2011;36:357–60. 22073888

[37] Ye Y Liu B. Analgesic effects of balanced acupuncture versus body acupuncture in low-back and leg pain patients with lumbar disc herniation, as assessed by resting-state functional magnetic resonance imaging. Neural Regen Res. 2012;7:1624–9. 25657702 10.3969/j.issn.1673-5374.2012.21.004 PMC4308764

